# Purification and SAXS Analysis of the Integrin Linked Kinase, PINCH, Parvin (IPP) Heterotrimeric Complex

**DOI:** 10.1371/journal.pone.0055591

**Published:** 2013-01-31

**Authors:** Amy L. Stiegler, Thomas D. Grant, Joseph R. Luft, David A. Calderwood, Edward H. Snell, Titus J. Boggon

**Affiliations:** 1 Department of Pharmacology, Yale University School of Medicine, New Haven, Connecticut, United States of America; 2 Hauptman-Woodward Medical Research Institute, Buffalo, New York, United States of America; 3 The University at Buffalo, The State University of New York, Department of Structural Biology, Buffalo, New York, United States of America; 4 Department of Cell Biology Yale University School of Medicine, New Haven, Connecticut, United States of America; 5 Yale Cancer Center, Yale University School of Medicine, New Haven, Connecticut, United States of America; University of Queensland, Australia

## Abstract

The heterotrimeric protein complex containing the integrin linked kinase (ILK), parvin, and PINCH proteins, termed the IPP complex, is an essential component of focal adhesions, where it interacts with many proteins to mediate signaling from integrin adhesion receptors. Here we conduct a biochemical and structural analysis of the minimal IPP complex, comprising full-length human ILK, the LIM1 domain of PINCH1, and the CH2 domain of α-parvin. We provide a detailed purification protocol for IPP and show that the purified IPP complex is stable and monodisperse in solution. Using small-angle X-ray scattering (SAXS), we also conduct the first structural characterization of IPP, which reveals an elongated shape with dimensions 120×60×40 Å. Flexibility analysis using the ensemble optimization method (EOM) is consistent with an IPP complex structure with limited flexibility, raising the possibility that inter-domain interactions exist. However, our studies suggest that the inter-domain linker in ILK is accessible and we detect no inter-domain contacts by gel filtration analysis. This study provides a structural foundation to understand the conformational restraints that govern the IPP complex.

## Introduction

Integrin adhesion receptors are an essential class of cell surface glycoproteins that mediate cell adhesion, migration and spreading by linking the extracellular matrix with the actin cytoskeleton. Integrin activation is regulated, in part, by the binding of adaptor and signaling proteins to the short integrin cytoplasmic tails. Once recruited, these proteins convert integrins to their high-affinity/active conformations, which in turn triggers cellular responses to cell adhesion such as cell migration, differentiation and survival [1]. An important cytoplasmic component localized to integrin receptors at focal adhesions is the heterotrimeric protein complex comprised of the integrin linked kinase (ILK), parvin, and PINCH, termed the IPP complex for its member proteins. The IPP complex is essential for focal adhesion formation, and serves as a hub for integrin and growth factor signaling to control cell adhesion, spreading and migration [2].

**Figure 1:**
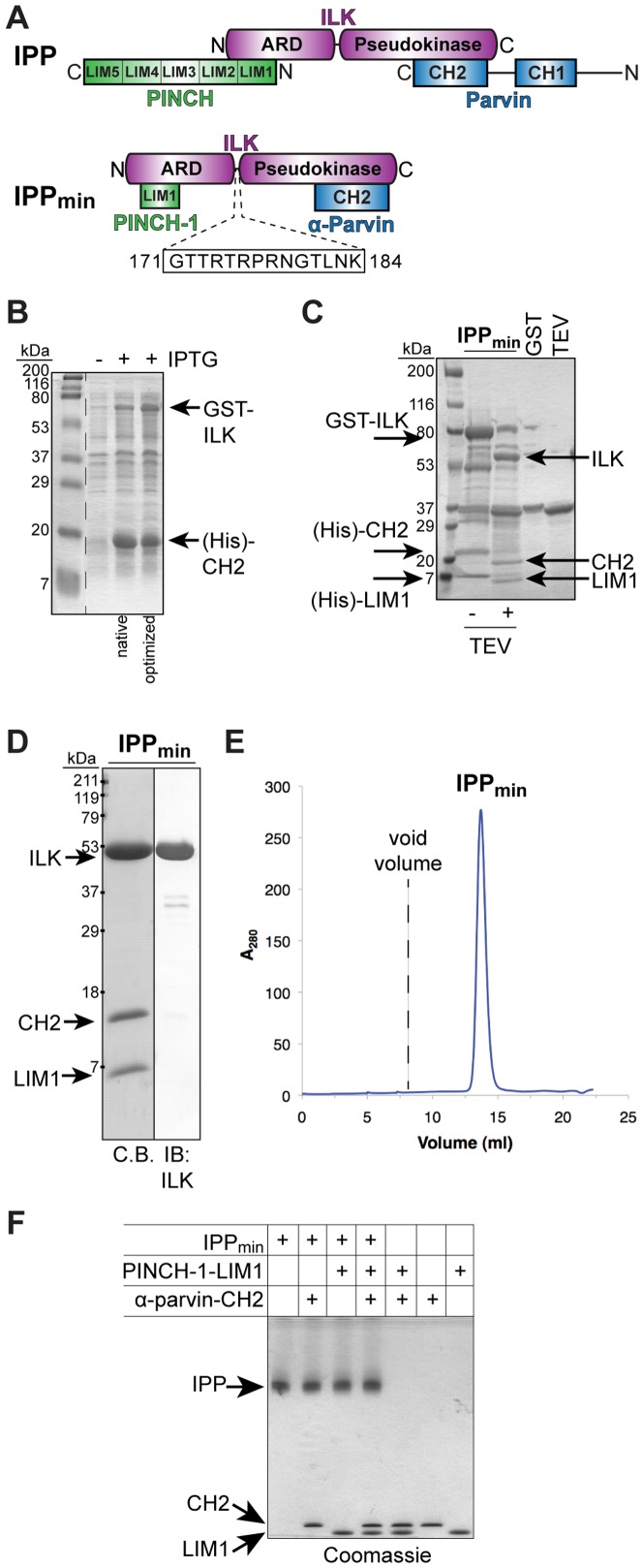
The IPP complex forms a stable, monodisperse, heterotrimeric complex. **A**) Schematic diagram of the IPP complex: Integrin-linked kinase (ILK; magenta), PINCH (green) and Parvin (blue). ILK is the hub of the complex, and binds the LIM1 domain of PINCH-1 via its N-terminal ankyrin-repeat domain (ARD), and the C-terminal calponin homology (CH2) domain of α-parvin via its C-terminal pseudokinase domain (pKD) to form the IPP_min_ complex. The 14 residue inter-domain linker in ILK is shown. The lengths of the proteins are drawn approximately to scale. **B**) Co-expression of GST-ILK and (His)-α-parvin-CH2 in *E. coli*. Codon-optimized cDNA encoding full-length human ILK shows increased expression relative to the native ILK cDNA. (His)-PINCH-1-LIM is expressed alone in *E. coli*. **C**) TEV proteolysis removes the GST- and (His)-tags. **D**) Purified IPP_min_ complex is resolved by SDS-PAGE and stained with Coomassie blue (C.B.) to show a high level of purity. Anti-ILK immunoblot confirms the presence of ILK in the complex. **E**) Gel-filtration chromatography of IPP_min_ reveals a monodisperse protein species. The elution volume is consistent with a monomeric protein complex. The void volume is indicated. **F**) Native gel electrophoresis of purified IPP_min_ indicates that IPP is a stable protein complex. Purified IPP_min_ protein alone, and IPP_min_ plus added excess PINCH-1-LIM1 and/or α-parvin-CH2 proteins are resolved by native gel electrophoresis and visualized by Coomassie blue staining.

ILK was first identified as an integrin β1 cytoplasmic tail binding protein [3], and is the central member of the IPP complex. In its N-terminus, five ankyrin repeat domains mediate direct interaction with the LIN-11/Isl1/MEC-3 (LIM)-domain containing protein PINCH1 (or the related isoform PINCH2) via the LIM1 domain [4]–[8] (**Figure 1A**). The C-terminus of ILK contains a pseudokinase domain (which we term ‘pKD’) that was the source of a lengthy controversy concerning its putative catalytic activity. Recent structural and structure-directed studies have resolved this controversy to show a lack of enzymatic competence [9], [10]. There is direct interaction between the ILK pseudokinase domain and the second of two tandem calponin homology (CH) domains that are present in the parvin family of proteins (α, β, and γ) [11]–[13] (**Figure 1A**). It was originally reported that ILK contains a short pleckstrin homology (PH) domain (residues 180–212) between the ARD and pKD regions [14]; however, subsequent structural studies revealed that the majority of this segment (residues 185–212) is integral to the pseudokinase fold [9].

The heterotrimeric IPP complex forms in the cytoplasm prior to cell adhesion [15] and is targeted to focal adhesions by several potential mechanisms, including ILK interaction with integrin tails [3] and parvin binding to the focal adhesion protein paxillin [13], [16], [17]. Formation of the IPP complex also serves to stabilize and protect its members from proteasomal degradation [18], [19]. Each individual component is critical for proper development, and a single deletion of either ILK, α-parvin or PINCH1 in mice causes embryonic lethality [20]–[23]. The IPP complex serves as a physical link between focal adhesion components, and interacts with a variety of proteins in the cytoplasm, including PINCH1 with Nck-2 [5], ILK with Kindlin-2 [24], [25] and the parvins with paxillin [13], [16], [17]. Furthermore, the IPP complex is implicated in several signaling pathways which include Akt/PKB, GSK3β/β-catenin, JNK, α-PIX/Rac1 [2], [26], [27].

In this study we present the first biochemical and structural analysis of the minimal heterotrimeric IPP complex. We provide a detailed purification protocol for IPP and show that the purified IPP complex is stable and monodisperse in solution. We then conduct SAXS-based structural characterization of the IPP complex and find that the averaged *ab initio* SAXS-derived molecular envelope is extended in shape with dimensions 120×60×40 Å. Flexibility analyses of the SAXS data support that the overall IPP complex exhibits limited flexibility, suggesting that inter-domain contacts exist. However, limited proteolysis indicates that the inter-domain linker in ILK is accessible, and gel filtration analysis reveals no measurable interaction between the N- and C-terminal domains. Our results support a model by which the minimal IPP complex adopts a predominantly compact conformation.

## Methods

### Expression

Synthetic cDNA encoding full-length ILK (UniProt Q13418 residues 1–452) codon-optimized for expression in *E. coli* was purchased from GenScript (Piscataway, NJ) and subcloned into a modified pET32 vector containing a TEV-cleavable GST tag and kanamycin resistance. cDNA encoding the CH2 domain of α-parvin (UniProt Q9NVD7 residues 242–372) was subcloned into the BamHI/XhoI sites of pCDFDuet-1 (Novagen), which carries Sterptomycin resistance. A TEV-cleavage sequence 5′ to the CH2-encoding region was added by PCR. The pET32 expression construct for His-tagged PINCH1-LIM1 (UniProt P48059, residues 6–68) was described previously [7], [8]. The GST-ILK and (His)-α-parvin-CH2 expression constructs were co-transformed into BL21(DE3) cells and grown under double selection in Kanamycin and Streptomycin. (His)-PINCH1-LIM1 was transformed into BL21(DE3) cells and expressed alone. Protein expression was induced at culture OD_600_ = 0.6–0.8 with 0.5 mM IPTG and conducted at 16°C for 18 h. Cells were harvested by centrifugation, resuspended in 15 ml lysis buffer (50 mM Tris pH 8.0, 150 mM NaCl) per L of culture, and mixed together prior to treatment with lysozyme (5 mg per L of culture), Complete Protease Inhibitor Tablet (Roche), 1 mM PMSF. Cells were then sonicated, and lysates treated with DNaseI, clarified by centrifugation and filtration, and supplemented with 1 mM DTT and 0.1% Triton-X 100.

### Protein Purification

Lysates were applied to glutathione-agarose 4B beads (GE Healthcare) at 4°C and collected by gravity flow. The flow-through sample was collected, and reapplied to the glutathione column a total of three times. The beads were washed three times with 10 column volumes (CV) of lysis buffer plus 1 mM DTT, and the column flow stopped before addition of freshly prepared elution buffer (15 mM reduced glutathione in lysis buffer, 1 mM DTT). Beads were incubated with elution buffer for 5 minutes, and the eluate collected. Elution was performed with 7–10 fractions of elution buffer, and the evaluated by SDS-PAGE. Elution fractions containing IPP complex were pooled. His-tagged recombinant TEV protease was added at a final concentration of 0.01–0.1 mg/ml and incubated overnight at 4°C, to remove the GST- and (His)-tags. The sample was then diluted for injection onto a 1 mL Mono Q column (GE Healthcare) to 50 mM Tris, pH 7.5, 30 mM NaCl, 1 mM DTT. A shallow gradient over 80 CV from 3 to 13% Buffer B (50 mM Tris pH 7.5, 1 M NaCl, 1 mM DTT) was applied in order to differentially elute GST from IPP protein, and 2 ml fractions collected. To remove remaining contaminating (His)-TEV protease and/or GST, the fractions containing IPP complex proteins (as determined by SDS-PAGE) were incubated with 50 µl of glutathione-agarose 4B plus 50 µl Ni-Agarose beads for 1 h at 4°C. The sample was then concentrated to 2 ml in a Centrifugal Filtration Unit (Millipore) and further purified by size-exclusion chromatography (Superdex 200 prep grade 16/60; GE Healthcare) equilibrated in 25 mM Tris, pH 7.5, 150 mM NaCl, 1 mM DTT. Fractions containing IPP proteins were pooled and concentrated to a final concentration of 7.0 mg/ml and filtered through a 0.22 µm filter. In general, 10 L of GST-ILK/(His)-α-parvin-CH2 plus 4 L PINCH-1-LIM1 yields 3 milligrams of the purified IPP protein complex. Western blotting for ILK was performed with anti-ILK antibody (#3862, Cell Signaling Technology). Native gel electrophoresis was performed on a PhastGel System (GE Healthcare). Limited trypsin proteolysis was performed at room temperature with serially diluted trypsin (Sigma 4799). Analytical size-exclusion chromatography (Superdex 200 10/300 GL; GE Healthcare) of full-length purified IPP_min_ and the trypsin proteolyzed complex was performed in 20 mM Tris, pH 7.5, 150 mM NaCl, 1 mM DTT.

### Small Angle X-ray Scattering

Solutions of IPP complex were prepared in buffer (25 mM Tris, pH 7.5, 150 mM NaCl, 1 mM DTT) at protein concentrations of 7.0, 5.2, 3.5, and 1.7 mg/ml. Scattering data were collected on beamline 4-2 at the Stanford Synchrotron Radiation Lightsource (SSRL). Data were collected on a MarCCD225 detector at a wavelength of 1.3 Å. 8 individual 1 sec exposures were collected for each concentration, with buffer scans collected before and after each experiment. Data were integrated and averaged with SasTool [28]. Buffer blanks were averaged and subtracted from the data. Each of the eight exposures was inspected visually in Primus [29] to ensure that there was no evidence of radiation damage and averaged using SasTool. Guinier analysis and radius of gyration (Rg) estimation were performed in Primus and confirmed by automatic analysis using AutoRG [29].

The pair distribution functions P(*R*) and forward scattering *I*(0) were computed automatically with AutoGNOM [30] and compared with those determined in Primusqt [29]. The molecular weights were estimated separately based on Porod volumes calculated in Primus [29], forward scattering *I*(0) of a reference solution of bovine serum albumin at 1.0 mg/ml, and excluded bead volumes of *ab initio* models calculated in DAMMIF [31]. Normalized (dimensionless) Kratky plots of *q*_n_^2^ vs. *I*_n_(*q*), where *q*_n_ = *q*×R_g_ and *I*_n_(*q*) = *I*(*q*)/*I*(0), were generated as described [32], [33]. For the highest IPP_min_ concentration (7.0 mg/ml), an angular range of *q*_min_ = 0.0161 Å^−1^ to *q*_max_ = 0.3417 Å^−1^ was chosen automatically by AutoGNOM. DAMMIF [31] was used to reconstruct ten *ab initio* envelopes using the P(*R*) from the highest concentration data and the molecular envelopes were averaged in DAMAVER [34]. During averaging, a mean normalized spatial discrepancy (NSD) value of 0.8 resulted from comparison of all ten models. DAMMIN [35] was used to further refine the averaged envelope.

Rigid body modeling was performed with CORAL [36] and flexibility analysis with the ensemble optimization method (EOM) [37], [38], using the crystal structures of human ILK-ARD/PINCH1-LIM1 (PDB accession code: 3F6Q) [8] (residues ILK: 1–170; PINCH1: 4–68, where residues 4 and 5 are vector-derived) and human ILK-pKD/α-parvin-CH2 (PDB accession code: 3KMU) [9] (residues ILK: 185–452; α-parvin: 246–372) as individual subunits. Residues present in the crystal structures but not present in our expression constructs were deleted for consistency. For EOM analysis, the 14 amino acid linker (residues 171–184; **Figure 1A**) was modeled with RANCH [37]. Data were cut off at *q*_max_ = 0.23 Å^−1^. Theoretical scattering curves for the two subunits were obtained with CRYSOL [39]. Superpositions of models were performed in SUPCOMB with no symmetry (P1) applied; superpositions did not exclude fits rotated by two-fold rotation [40]. Porod-Debye analysis was performed, with scattering data in the range of *q*_min_ = 0.1222 Å^−1^ to *q*_max_ = 0.156 Å^−1^ plotted as *q* ^4^×*I*(*q*) vs *q* ^4^ used for linear fitting [33].

## Results

### Purification of a minimal IPP complex

For biochemical, biophysical and crystallographic experiments, we established a bacterial expression system and purification scheme for a minimal IPP complex (IPP_min_) containing ILK, PINCH1 and α-parvin proteins. As shown previously, the first LIM domain (LIM1) of PINCH1 is sufficient to bind the N-terminal ankyrin-repeat domain of ILK (ILK-ARD) [4]–[6], and the C-terminal calponin homology (CH2) domain of α-parvin is sufficient to bind the C-terminal pseudokinase domain of ILK (ILK-pKD) [11]–[13] (**Figure 1A**). ILK expression constructs containing the pseudokinase domain are largely insoluble when expressed alone in *E. coli*; however, co-expression with α-parvin-CH2 results in a soluble protein complex [[9] and data not shown]. Therefore, we co-expressed full-length ILK as a GST-fusion protein and α-parvin-CH2 as a His-tagged protein in *E. coli* by co-transformation of compatible expression vectors under dual selection, which rescues ILK protein from insolubility (data not shown). Codon-optimized synthetic cDNA encoding ILK showed improvement of total yield over native cDNA, and was used in subsequent purifications (**Figure 1B**). His-tagged PINCH1-LIM1, which is soluble in *E. coli*, was expressed alone, and cells mixed with the ILK/α-parvin-CH2 expressing cells prior to co-sonication. The IPP_min_ complex was affinity purified on glutathione-agarose beads, eluted, and the GST- and (His)- tags simultaneously removed by TEV proteolysis (**Figure 1C**). IPP_min_ complex was then purified by anion exchange chromatography using a shallow gradient, which allowed for differential elution of GST and IPP_min_. Remaining GST protein was then removed by incubation with glutathione-agarose beads and size-exclusion chromatography to achieve purity of crystallization quality, and immunoblot analysis confirms the presence of ILK in the complex (**Figure 1D**). Notably, the IPP_min_ complex remains intact during all steps of purification. The migration of ILK, α-parvin-CH2 and PINCH-1-LIM1 on SDS-PAGE is consistent with their expected molecular weights (51.4 kDa, 14.7 kDa, and 7.5 kDa, respectively). Purified IPP_min_ protein resolved on a calibrated gel-filtration column results in a single peak with an elution volume corresponding to a molecular weight of approximately 80 kDa, in close agreement with the expected molecular weight of 73.6 kDa (**Figure 1E**). The purified IPP_min_ complex migrates as a single species on native gel electrophoresis and its migration is unchanged upon addition of excess purified PINCH1-LIM1 and/or α-parvin-CH2 proteins, confirming that the purified IPP_min_ complex is stable and monodisperse in solution (**Figure 1F**).

### SAXS analysis of IPP_min_ complex

**Figure 2:**
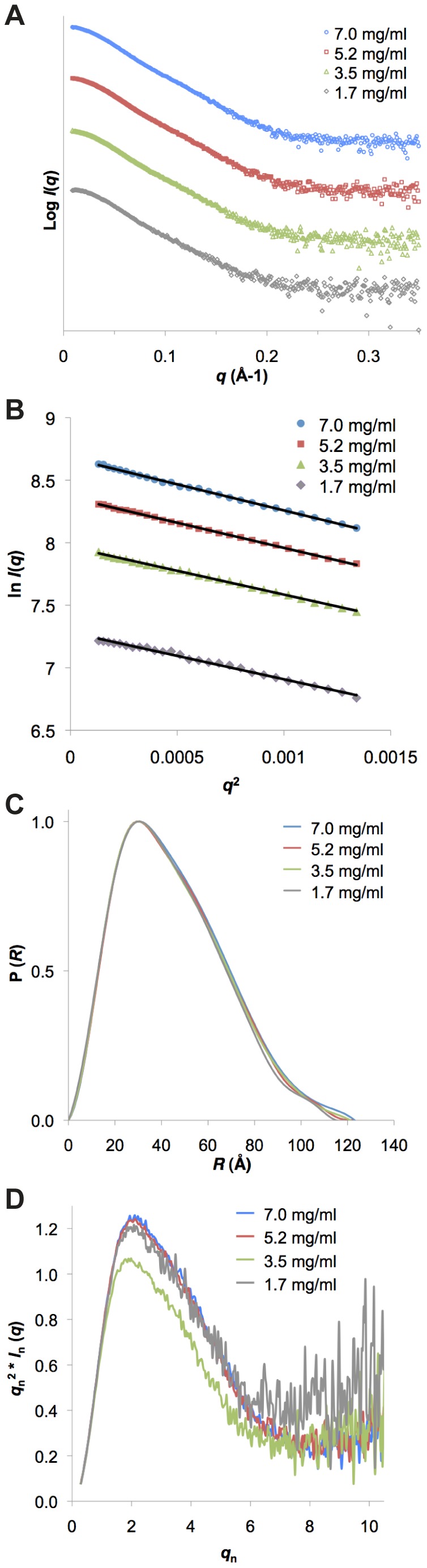
SAXS analysis for IPP_min_ reveals a globular heterotrimeric complex. **A**) SAXS intensity profiles (logarithmic) for four concentrations of the IPP_min_ complex. **B**) Linearity of Guinier plots with manual selection of Guinier region. The *R*_g_ values are presented in **Table 1**. Automatic Guinier analysis performed in AutoRG [29], which is consistent with the analysis shown here, is presented in the Supporting Information. **C**) Normalized pair distribution functions P(*R*) calculated automatically with AutoGNOM [30]. **D**) Dimensionless Kratky plots support a globular shape.

To study the overall conformation of the IPP in solution, we collected SAXS data on a concentration series of IPP_min_ complex protein (**Figure 2A**). First, we analyzed the SAXS intensity data to confirm that IPP_min_ protein is monodisperse and monomeric in solution. We find that Guinier plots are linear in the Guinier region (q*R_g_<1.3) (**Figure 2B**) and that Guinier approximation for the radius of gyration (*R*_g_) values are consistent for all of the IPP_min_ concentrations measured, ranging from 33.9 Å to 35.3 Å (**Table 1**), confirming a lack of aggregation in the sample. Automatic generation of Guinier plots using AutoRg [29] results in similar *R*_g_ values (**Figure S1 and Table S1**). Further evidence to support the lack of aggregation comes from the maximum particle dimension (D_max_) for each of the concentrations which are in close agreement with one another and range from 120.0 Å to 123.2 Å (**Table 1**). Next, the molecular weights based on Porod volumes and forward scattering were computed and compared with the expected molecular weight of the IPP_min_ complex (**Table 1**). Taken together with the elution profile from size-exclusion chromatography (**Figure 1E**), we conclude that the IPP_min_ protein complex is monodisperse and monomeric in solution. The pairwise distribution functions, P(*R*), which reflects the inter-atomic distance distributions, were then determined, and are very similarly shaped for each of the IPP_min_ concentrations (**Figure 2C**). The asymmetric P(*R*) functions, which tail-off at higher *q* values, are consistent with a slightly elongated molecule with an asymmetric shape. The P(*R*) functions peak around 35 Å with a small shoulder around 50 Å, potentially indicating a second structural unit. Dimensionless Kratky plots show the characteristic globular peak for a folded protein (**Figure 2D**). Since no aggregation or repulsion is evident in the samples and since consistent *R*_g_, D_max_ and molecular weight values (**Table 1**) indicate no conformational change with concentration, ‘zero extrapolation’ was not performed and data from the highest concentration of IPP_min_ (7.0 mg/ml) was used for all subsequent analysis.

**Table 1:** SAXS-derived size parameters for IPP_min_.

Concentration (mg/ml)	*R*_g_ a	*R*_g_ b	D_max_	Molecular Mass (Da)^c^		
				Porod	*I*(0)	Excluded volume
7.0 (95.1 µM)	35.3±0.1	35.8	123.2	72,568	78,983	72,289
5.2 (71.3 µM)	34.7±0.1	35.3	120.3	72,478	77,218	69,880
3.5 (47.6 µM)	33.9±0.1	35.3	120.0	69,888	79,631	70,482
1.7 (23.8 µM)	33.9±0.2	34.6	121.5	68,131	79,901	69,880

a Determined by Guinier approximation in Primus [29].

b Determined in AutoGNOM [30].

c Expected molecular weight = 73,625 Da.

### SAXS-based structural modeling of IPP

**Figure 3:**
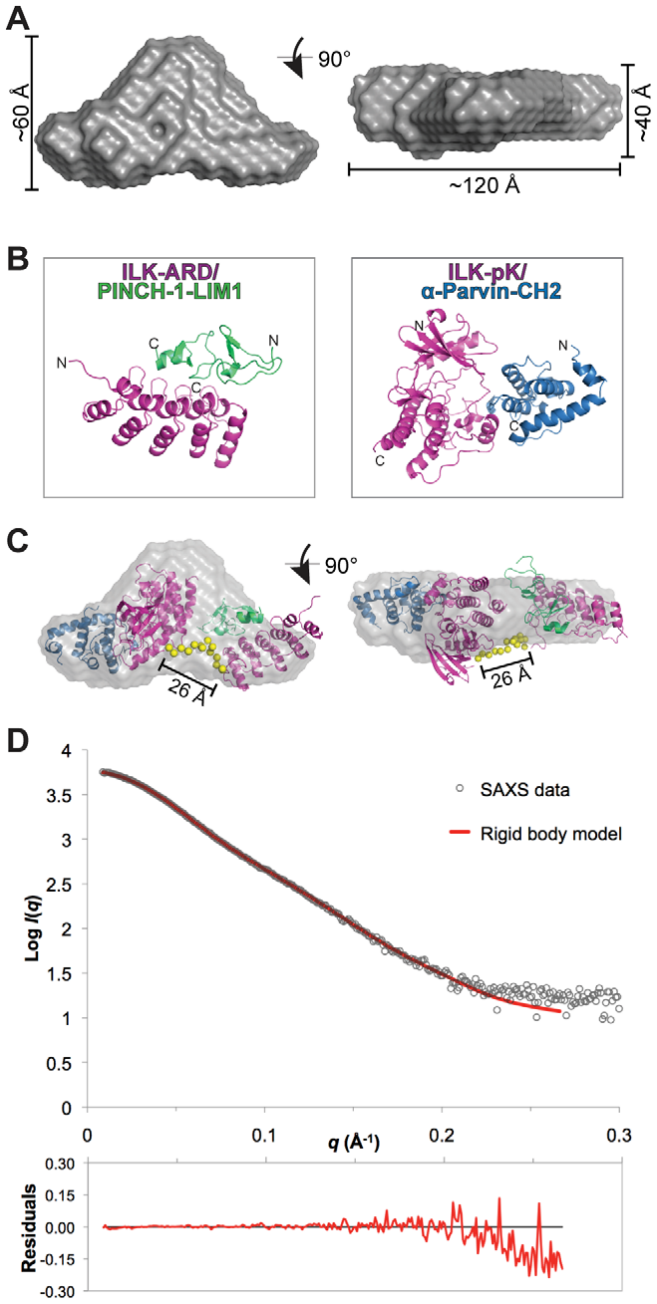
Structural modeling of IPP_min_ based on SAXS data. **A**) Averaged molecular envelope for IPP_min_. The approximate envelope dimensions (in Å) are illustrated. The two views are related by 90° rotation. **B**) The crystal structures of the individual subunits of the IPP_min_ complex, ILK-ARD/PINCH-1-LIM1 (PDB code: 3F6Q) and ILK-pseudokinase (pKD)/α-parvin-CH2 (PDB code: 3KMU) used in rigid body modeling. ILK is colored magenta, PINCH-1 is green, and α-parvin is blue. **C**) CORAL [36] rigid body model of IPP_min_ (ribbons, colored as in **B**) with the best statistical fit to the experimental data (plotted in **D**). Overlaid is the averaged molecular envelope. 14 inter-domain dummy residues between the C-terminus of ILK-ARD and the N-terminus of ILK-pKD, in the optimal conformation chosen by CORAL, are depicted as yellow spheres. The distance between the two subunits is 26 Å. **D**) Fit of the theoretical scattering profile for the rigid body model (red line) with the experimental SAXS data (logarithmic). Residuals for the fit are shown below.

We performed structural modeling of the SAXS data using two different approaches. Using the P(*R*) function, ten individual *ab initio* molecular envelopes (dummy beads models) were reconstructed and averaged. The averaged envelope reveals a slightly extended shape that resembles a bicorne hat (**Figure 3A**) with dimensions 120×60×40 Å, consistent with the experimentally determined R_g_ and D_max_ values (**Table 1**). The envelope is asymmetric on its long axis, with one end slightly larger than the other. We next conducted rigid body modeling of the two subunits of IPP_min_ with CORAL [36]. Based on the protein boundaries in the available crystal structures versus our full-length ILK construct, the un-modeled linker between the ILK-ARD and ILK-pKD subunits is 14 residues (residues 171–184; **Figure 1A**). In rigid body analysis, the relative orientation between ILK-ARD/PINCH1-LIM1 and ILK-pKD/α-parvin-CH2 (**Figure 3B**) was refined by simulated annealing using a pre-calculated library of random, self-avoiding loops containing 14 dummy residues to constrain the distance between the two subunits, in order to best fit the experimental scattering data. This results in a model of IPP_min_ with overall shape similar to the averaged molecular envelope, with an inter-domain distance of approximately 26 Å (**Figure 3C**). The rigid body model fits well with the experimental data, with a χ value of 1.4 (**Figure 3D**).

### IPP_min_ adopts a predominantly compact conformation in solution

**Figure 4:**
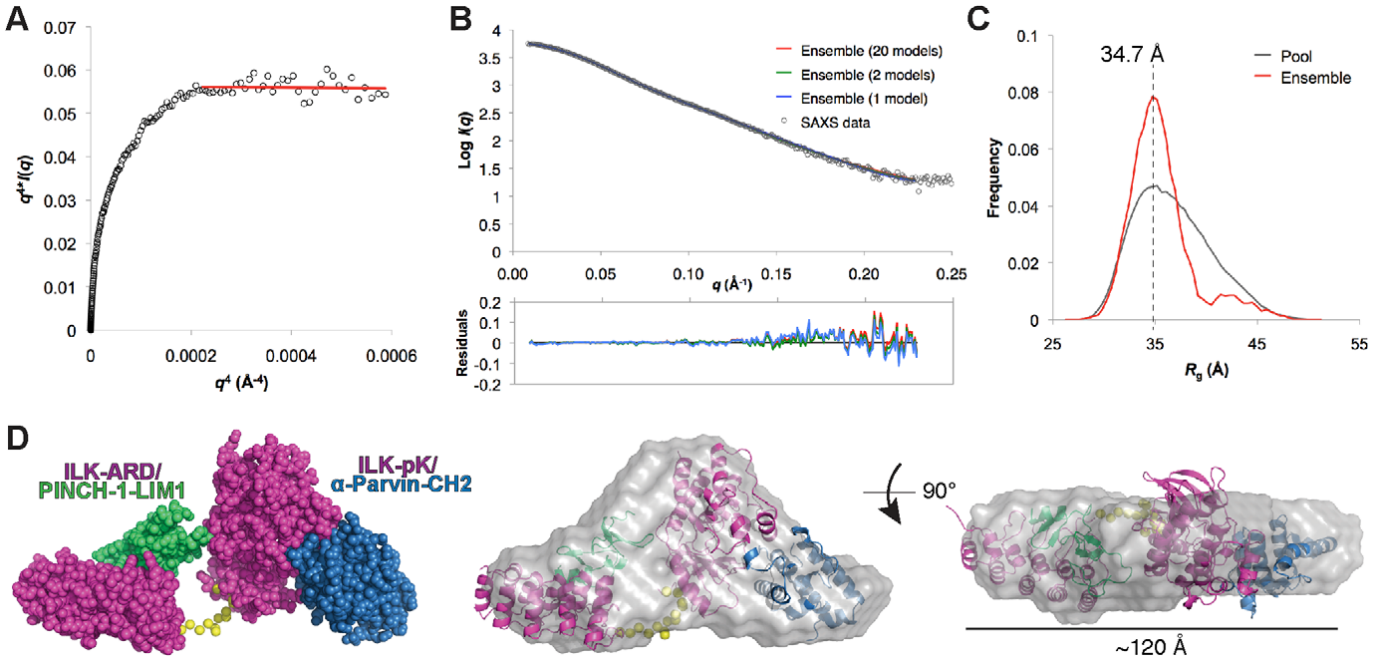
Flexibility analysis of IPP_min_. **A**) Porod-Debye plot of IPP_min_ SAXS data (open circles) shows a linear plateau (red line) consistent with a folded, globular protein with little flexibility. **B thru D**) Ensemble Optimization Method (EOM). **B**) Fits of the theoretical scattering profiles for the selected ensembles (containing 20 models: red line, 2 models: green line, 1 model: blue line) with the experimental SAXS data (logarithmic scale; top) and the residuals of the fits (bottom). **C**) *R*_g_ size distribution (Å) for selected ensemble (20 models, red) compared with the pool of 10,000 models (grey) used for EOM showing two populations of IPP_min_ structures. The dashed line represents the average *R*_g_ value (34.7 Å) of the predominant conformation in the optimized ensemble. **D**) EOM-generated model representing the most representative structure of IPP in the optimized ensemble (NSD = 1.3), which is overlaid with the averaged molecular envelope. The D_max_ of the respective models is shown. Protein domains are labeled and colored as in Figure 3B.

We next assessed inter-domain flexibility in the IPP_min_ complex. A Porod-Debye plot of the IPP_min_ scattering data (**Figure 4A**) shows a plateau that fits the linear plot with a Porod Exponent of 4, consistent with a well-ordered, globular particle with little to no flexibility [33]. We also examined flexibility using the ensemble optimization method (EOM) [37]. The ILK-ARD/PINCH1-LIM1 and ILK-pKD/α-parvin-CH2 structures were treated as rigid bodies connected by a flexible linker of 14 dummy residues missing from the crystal structures (residues 171–184; **Figure 1A**), and a pool of 10,000 individual models were generated containing a random sampling of linker conformations and subunit positions avoiding steric clashes to cover the range of configurational space. A genetic algorithm was then employed to select an optimized ensemble of models whose combined theoretical scattering curve best represents the IPP_min_ experimental scattering profile. EOM analysis of our IPP_min_ SAXS data yields an optimized ensemble (containing 20 models) that fits the experimental scattering curve with *χ* value of 1.5 (**Figure 4B**). An ensemble containing as few as 2 models fits the experimental data equally well as the larger 20 model ensemble (*χ* = 1.5, **Figure 4B**), suggesting limited flexibility. A single model ensemble, in contrast, fits the data slightly less well, with *χ* = 1.8 (**Figure 4B**), suggesting the potential for a small population of a second conformation of IPP_min_. However, since the fit to the experimental data of the rigid body model (χ = 1.4, Figure 3) is as good as the EOM optimized ensemble (*χ* = 1.5), our data support a structure in which IPP_min_ exhibits limited flexibility.

Comparing the *R*_g_ distributions of the optimized ensemble with the random pool, we find that the ensemble displays a more narrowed *R*_g_ distribution, with a major peak at *R*_g_ = 34.7 Å (**Figure 4C**). This is in good agreement with the value calculated from the scattering curve (**Table 1**), and represents a predominant, compact IPP_min_ conformation in solution. We also observe a second minor, broad *R*_g_ peak above 40 Å, which may indicate the presence of a small fraction of more extended IPP_min_ particles in solution (models with Rg above 40 Å are selected at a frequency of 10% in the optimized ensemble). When repeating EOM analysis with SAXS data collected on lower IPP_min_ concentrations, we find that the trend in *R*_g_ distributions is largely unaffected by concentration (not shown) suggesting that the more elongated particle does not represent a concentration-dependent aggregate of IPP_min_. However, we cannot exclude the possibility that the small peak at higher *R*_g_ values is an artifact of modeling and/or over-fitting of the high angle scattering data, or that a small percentage of IPP_min_ forms aggregates in all concentrations measured.

We next assessed the structural variability in the selected ensemble by superposition using normalized spatial discrepancy (NSD) values [38]. The optimized ensemble has a NSD value of 1.4±0.1, lower than the NSD value for a set of 100 randomly-chosen conformers from the pool (NSD = 1.6±0.2), consistent with a predominant IPP_min_ particle in solution. The most representative model from the optimized ensemble, which shows the smallest average variation (NSD = 1.3), adopts a somewhat compact shape that fits well with the molecular envelope (**Figure 4D**). This model has a *R*_g_ value of 35.4 Å and D_max_ of 128.7 Å, consistent with values calculated from the scattering curve (Table 1). Taken together, the results from EOM analysis support that IPP_min_ adopts a predominantly compact structure in solution with limited flexibility.

### ILK contains an unstructured inter-domain linker

**Figure 5:**
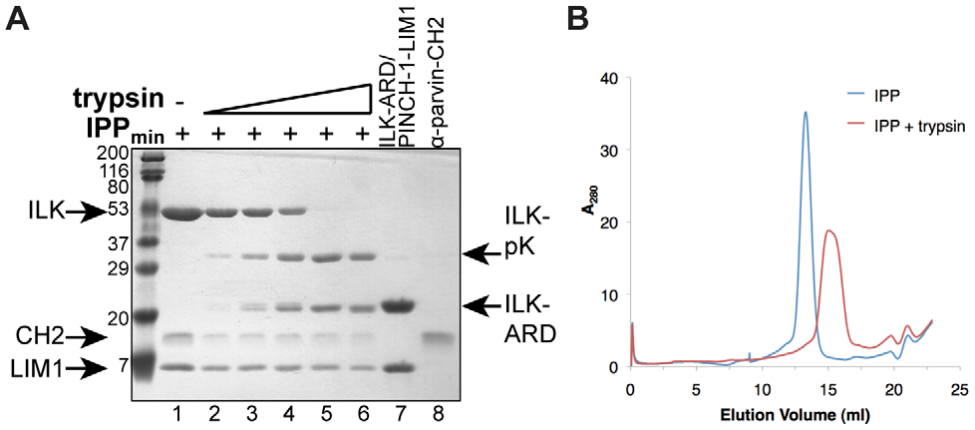
An unstructured linker in ILK connects the N- and C-terminal subunits of IPP. **A**) Limited trypsin proteolysis of purified IPP_min_ complex (lanes 2 through 6) supports that the linker in ILK is unstructured. The N-terminal IPP subunit (ILK-ARD/PINCH-1-LIM1, lane 7) and α-parvin-CH2 alone (lane 8) are included for comparison. Molecular weight markers (in kDa) are shown. **B**) Gel-filtration chromatography of the full-length IPP_min_ protein (lane 1 from part D) and trypsin proteolyzed subunit fragments (lane 6 from part D) reveals no apparent interaction between the N- and C-terminal subunits of the IPP complex.

The N-terminal ILK-ARD and C-terminal ILK-pKD subunits are separated by a 14-residue linker (**Figure 1A**) that sequence profile analysis suggests is unstructured/disordered (PSIpred [41], DISOPRED [42], PrDOS [43], DisEMBL [44], data not shown). From EOM analysis, the predominant IPP_min_ structure is somewhat compact (**Figure 3B** **and** **4C**), with D_max_ values consistent with an average inter-subunit linker of approximately 25 Å. Similarly, rigid body modeling results in a linker of approximately 19 Å. Considering that a fully extended linker could be as long as 50 Å, this shorter average distance raises the possibility that the linker contains secondary structure and/or is partially structured through interactions with either the N-terminal ARD or C-terminal pKD of ILK. We therefore probed disorder in the ILK inter-domain linker by subjecting the purified IPP_min_ complex to limited trypsin proteolysis. As shown in **Figure 5A**, IPP_min_ is easily proteolyzed into its two subunits; once cleaved, the domains appear resistant to further proteolysis, suggesting that they are stable structural units. This result indicates that the 14-residue linker in ILK, which contains 3 predicted trypsin cleavage sites (Arg-174, Arg-178, Lys-184, **Figure 1A**), is at least partially exposed in solution and unstructured.

Our finding of a major, compact IPP_min_ conformation also suggests that inter-domain interactions between the N- and C-terminal subunits of IPP_min_ could occur (**Figure 3B** **and** **4E**). To test this possibility, we resolved full-length IPP_min_ or the trypsin proteolyzed fragments on gel filtration chromatography. Based on the relative elution volumes (**Figure 5B**), we do not detect inter-domain interaction between the N-terminal ILK-ARD/PINCH1-LIM1 and C-terminal ILK-pKD/α-parvin-CH2 subunits of IPP. Taken together, our structural analysis supports a model in which, connected by a partially unstructured linker, the N-terminal ILK-ARD/PINCH1-LIM1 and C-terminal ILK-pKD/α-parvin-CH2 subunits of IPP are not strongly fixed by strong inter-domain interactions. However, it remains possible that weaker inter-domain interactions serve to stabilize the predominant conformation of IPP detected in SAXS flexibility analysis.

## Discussion

The heterotrimeric IPP protein complex is a critical cytoplasmic component localized at integrin-rich focal adhesions [2]. Complex formation is a critical step in the functions of IPP: it occurs prior to and is important for correct focal adhesion targeting of its member proteins [15] and it serves to stabilize and protect its member proteins from degradation [18]. Our biochemical studies of the purified IPP complex, along with previous reports of the individual subunits, strongly suggest that the minimal binding fragments interact with high affinity and form stable complexes in solution (**Figure 1** and [7]–[10], [45]). Furthermore, previous investigations into the complex as a whole are consistent with the IPP being an interdependent entity for function of its member proteins ILK, PINCH and parvin, in their roles of focal adhesion maturation and muscle adhesion [19], [46]. Thus, the heterotrimeric IPP complex containing ILK, PINCH1 and α-parvin may be considered a single, stable structural and functional unit. Similarly, distinct IPP complexes containing PINCH2, β-parvin or γ-parvin, which compete with PINCH1 and α-parvin, are also expected to be stable [7], [47], [48].

Here, we show by SAXS analysis that the IPP complex comprised of full-length ILK and the minimal binding domains from PINCH1 and α-parvin forms a predominantly compact structure in solution (**Figure 4**). This raises the possibility that inter-domain contacts between the N- and C-terminal domains of IPP could serve to stabilize the relative orientations of the two subunits, allowing the compact structure to be the major IPP species. However, we do not detect a measurable interaction between the two IPP subunits in our gel filtration studies (**Figure 5**). Nonetheless, it remains plausible that weaker, transient inter-domain contacts exist in an intact IPP complex. These may take the form of a direct interaction in *cis* between the ARD and pKD subunits of ILK, between ILK-ARD/α-parvin-CH2 or ILK-pKD/PINCH1-LIM1, or between α-parvin-CH2 and LIM1. Additional studies will be required to carefully assess potential low-affinity interactions between the IPP subunits.

There are several potential functional implications of inter-domain contacts within the IPP complex. Inter-domain interactions could represent an autoinhibited state in which binding partner sites are occluded by inter-domain interaction. Since the IPP subunits are flexible relative to one another, this autoinhibition would be transient, allowing short-term access to a binding surface that would then be stabilized. We note that phosphorylation of ILK at Thr-173, within the unstructured linker of ILK, has been demonstrated [49], potentially presenting a mechanism by which the linker could stabilize inter-domain interaction in the cell. Alternatively, inter-domain contacts within IPP could provide a contiguous binding site for a binding partner when properly aligned. However, it does not appear that IPP is pre-aligned for a binding event involving a contiguous surface, since we detect some flexibility in IPP. ILK reportedly interacts directly with integrin β-tails and kindlin [3], [25], PINCH1 binds Nck-2 [50], and α-parvin binds paxillin and F-actin [16], [51]. It will therefore be interesting to see whether these and other binding events are associated with distinct conformational states of the IPP complex.

## Supporting Information

Figure S1 **Automatic Guinier Analysis.** Linear region of the Guinier plots as determined automatically by AutoRG (Primus) [29]. The *R*_g_ values are presented in Table S1.
(TIFF)

Table S1 ***R*** **_g_ values determined by automatic Guinier Analysis in AutoRG** [**29**] **.**
(DOC)
