# Supplementary figures and images for: Purification and SAXS Analysis of the Integrin Linked Kinase, PINCH, Parvin (IPP) Heterotrimeric Complex
Source: PLoS One. 2013 Jan 31;8(1):e55591. doi: 10.1371/journal.pone.0055591 (PMC3561323; doi:10.1371/journal.pone.0055591)

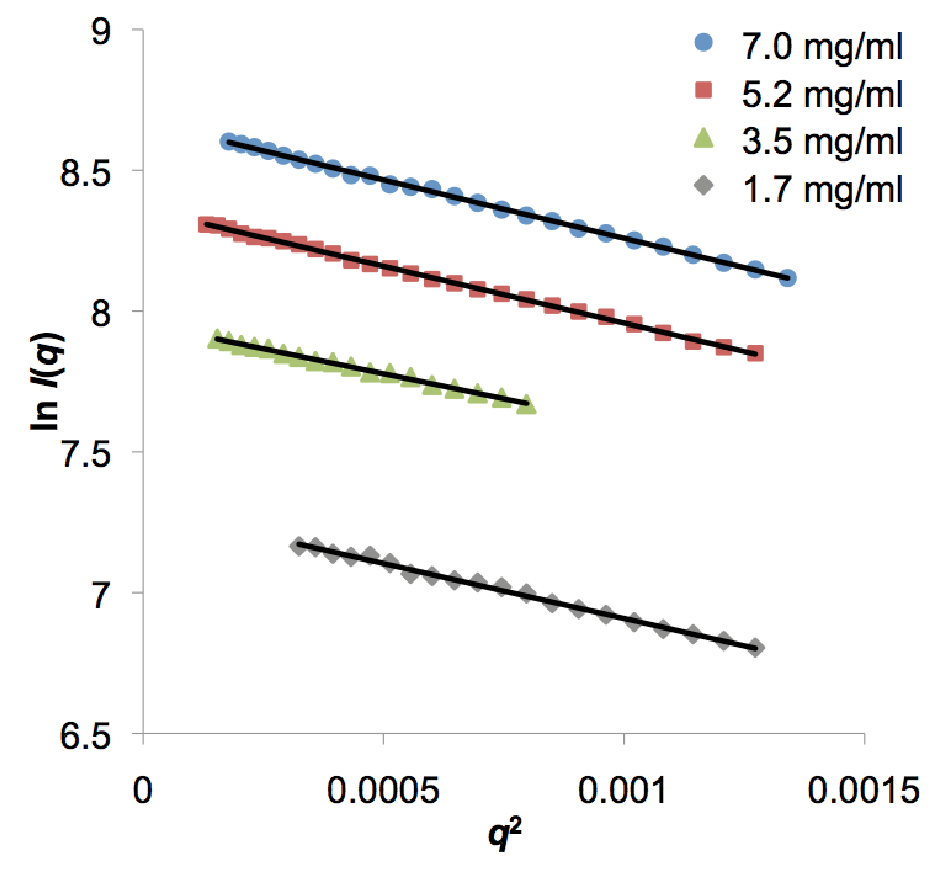

Supplement: Figure S1 — Automatic Guinier Analysis. Linear region of the Guinier plots as determined automatically by AutoRG (Primus) [29]. The R g values are presented in Table S1. (TIFF) [file pone.0055591.s001.tiff]
