# Supplementary material for: Purification and SAXS Analysis of the Integrin Linked Kinase, PINCH, Parvin (IPP) Heterotrimeric Complex
Source: PLoS One. 2013 Jan 31;8(1):e55591. doi: 10.1371/journal.pone.0055591 (PMC3561323; doi:10.1371/journal.pone.0055591)
Supplement: Table S1 — R g values determined by automatic Guinier Analysis in AutoRG [29] . (DOC) [file pone.0055591.s002.doc]

| Concentration  (mg/ml) | *R*_g_ (AutoRG) |
| --- | --- |
| 7.0 (95.1 μM) | 35.2 ± 0.3 |
| 5.2 (71.3 μM) | 34.8 ± 0.3 |
| 3.5 (47.6 μM) | 32.6 ± 1.5 |
| 1.7 (23.8 μM) | 34.2 ± 1.9 |
